# Periplocymarin Induced Colorectal Cancer Cells Apoptosis *Via* Impairing PI3K/AKT Pathway

**DOI:** 10.3389/fonc.2021.753598

**Published:** 2021-11-25

**Authors:** Yi Cheng, Guiying Wang, Lianmei Zhao, Suli Dai, Jing Han, Xuhua Hu, Chaoxi Zhou, Feifei Wang, Hongqing Ma, Baokun Li, Zesong Meng

**Affiliations:** ^1^ Department of Dermatology, The Fourth Hospital of Hebei Medical University, Shijiazhuang, China; ^2^ Department of General Surgery, The Fourth Hospital of Hebei Medical University, Shijiazhuang, China; ^3^ Department of Gastrointestinal Surgery, The Third Hospital of Hebei Medical University, Shijiazhuang, China; ^4^ Scientific Research Center, The Fourth Hospital of Hebei Medical University, Shijiazhuang, China; ^5^ Department of Medical Oncology, The Fourth Hospital of Hebei Medical University, Shijiazhuang, China

**Keywords:** colorectal cancer, periplocymarin, apoptosis, IRS1, PI3K/AKT pathway

## Abstract

Colorectal cancer (CRC) is one of the most common cancers worldwide, and approximately one-third of CRC patients present with metastatic disease. Periplocymarin (PPM), a cardiac glycoside isolated from *Periploca sepium*, is a latent anticancer compound. The purpose of this study was to explore the effect of PPM on CRC cells. CRC cells were treated with PPM and cell viability was evaluated by CCK-8 assay. Flow cytometry and TUNEL staining were performed to assess cell cycle and apoptosis. Quantitative proteomics has been used to check the proteins differentially expressed by using tandem mass tag (TMT) labeling and liquid chromatography–tandem mass spectrometry. Bioinformatic analysis was undertaken to identify the biological processes that these differentially expressed proteins are involved in. Gene expression was analyzed by western blotting. The effect of PPM *in vivo* was primarily checked in a subcutaneous xenograft mouse model of CRC, and the gene expression of tumor was checked by histochemistry staining. PPM could inhibit the proliferation of CRC cells in a dose-dependent manner, induce cell apoptosis and promote G0/G1 cell cycle arrest. A total of 539 proteins were identified differentially expressed following PPM treatment, where among those there were 286 genes upregulated and 293 downregulated. PPM treatment caused a pro-apoptosis gene expression profile both *in vivo* and *in vitro*, and impaired PI3K/AKT signaling pathway might be involved. In addition, PPM treatment caused less detrimental effects on blood cell, hepatic and renal function in mice, and the anti-cancer effect was found exaggerated by PPM+5-FU combination treatment. PPM may perform anti-CRC effects by promoting cell apoptosis and this might be achieved by targeting PI3K/AKT pathway. PPM might be a safe and promising anti-cancer drug that needs to be further studied.

## Introduction

Global cancer statistics for 2020 showed that colorectal cancer (CRC) ranks third in terms of cancer incidence, but second in terms of cancer mortality, where more than 1.9 million new CRC cases and 935,000 deaths were estimated to occur worldwide (1). In developed countries, five-year survival of patients with CRC has been improved due to early screening, however, up to 25% patients still present with stage 4 disease, while 25 to 50% present with early-stage disease and subsequently go on to develop metastatic disease (2, 3). The prognosis for patients with metastatic CRC remains poor, with a median five-year survival of CRC in 2012–2015 just 56.9% in China (4). Chemotherapeutic agents such as FOLFOX, the main therapy strategy for CRC patients, are effective but combined with unwanted toxicity and side effects (5). Therefore alternative treatment options are urgently required.

To date, about 85% of all approved small-molecule anticancer drugs are from natural products directly or indirectly (6). Periplocymarin (PPM) is isolated from *cortex periplocae*, the dry root of *Periploca sepium* Bge, which is traditionally used as antirheumatic and diuretic agent in Chinese medicine (7). Previous studies have indicated that, PPM performed anti-tumor effects in various of cancer (8, 9). For example, it has been found that PPM promoted prostate adenocarcinoma (PC3) cell apoptosis and inhibit proliferation of U937, HCT-8, Bel-7402, BGC823, A549, and A2780 cell lines *in vitro* with IC50 values of 0.02–0.29 mM (10). Recently, PPM has been shown to exhibit the advantages of quick effect, short duration, and no accumulation (11). Furthermore, PPM is highly permeable with absence of P-glycoprotein efflux and cytochrome P450, indicating it is a potential natural compound for drug development (12). However, the effect of PPM on CRC has been seldom performed. In the present study, we have investigated the effect of PPM on CRC, to provide more candidate drugs for CRC patients and prolong their lives.

## Materials and Methods

### Cell Culture

The CRC cell lines HCT 116, SW480, RKO, and HT-29 were purchased from Type Culture Collection of the Chinese Academy of Science (Shanghai, China) (catalogue numbers were TCHu 99, TCHu172, TCHu116, and TCHu103, respectively) and preserved by the Scientific Research Center of the Fourth Hospital of Hebei Medical University. All cells were cultured in DMEM (Gibco Invitrogen, Grand Island, NY, USA) supplemented with 10% heat-inactivated fetal bovine serum (FBS) (Gibco Invitrogen, Grand Island, NY, USA), penicillin (100 U/ml), and streptomycin (100 μg/ml) (Invitrogen, Carlsbad, California, USA) and incubated at 37°C in air containing 5% CO_2_.

### CCK-8 Assay

PPM (purity ≥98%) was obtained from Tauto Biotechnology Co., Ltd (catalogue number E-2462, Shanghai, China). PPM was dissolved in DMSO and diluted to 12.5, 25, 50, or 100 ng/ml using DMEM medium (final concentration of DMSO <0.01%). Cells (1 × 10^4^ cells/well) were exposed to different concentrations of PPM (12.5, 25, 50, or 100 ng/ml) for 24 and 48 h separately. Cells with 0.01% DMSO but no PPM treatment were set as DMSO group. Cells without either PPM or DMSO treatment were set as control. Cell Counting Kit 8 (CCK-8) assay was performed to detect the proliferative ability of CRC cell lines according to the manufacturer’s instructions. Briefly, 10 μl of CCK-8 solution ((Meilun Biotechnology, Dalian, China) was added to each well by incubation for 2 h at 37°C in the dark. The absorbance was measured at 450 nm using spectrophotometer. The proliferation rate was calculated by the formula: proliferation rate = (As − Ab)/(Ac − Ab), in which As represents the test group, Ac the negative control group, and Ab the blank control group.

According to the results, HCT 116 and RKO cells treated with PPM (12.5, 25, 50, or 100 ng/ml) for 24 h were used for apoptosis and cell cycle analysis.

### Cell Apoptosis Detection

Cellular apoptosis was quantified by both flow cytometry and TUNEL staining kit. Briefly, approximately 5 × 10^5^ cells were harvested and suspended in 500 μl of binding buffer containing Annexin V and propidium iodide (PI) (both 5 μl) (BD Biosciences, San Diego, CA, USA). Then cells were incubated and analyzed by Annexin V-FITC/PI Staining kit according to the manufacturer’s instruction.

TUNEL staining was performed by using kit (Roche Diagnostic GmbH, Penzberg, Germany) following the manufacturer’s protocol. Cells were fixed by 4% paraformaldehyde and washed twice with PBS, and then incubated in solution containing 10 μl of 0.1% Triton X-100 (Sigma-Aldrich, USA) and 10 ml of 0.1% sodium citrate for 2 min. After drying, the TUNEL staining reaction solution was added to the cells, which was followed by 60 min incubation at 37°C in the dark. The cells were observed under a fluorescence microscope. Cells with 0.01% DMSO but no PPM treatment were set as control group.

### Cell Cycles Detection

Cell cycles were also determined by flow cytometry. Approximately 1 × 10^6^ cells were harvested and suspended with 1 ml of DNA staining solution and 10 μl of permeabilization solution (MultiSciences Biotech, Hangzhou, China). Cells were incubated at room temperature in the dark for 30 min and then analyzed.

### Liquid Chromatography–Mass Spectrometry/Mass Spectrometry Analysis

Proteins were extracted from HCT 116 cells treated by 50 ng/ml PPM for 24 h and untreated cells respectively by SDT [4% (*w*/*v*) SDS, 100 Mm Tris/HCL pH 7.6, 0.1 M DTT] lysis and quantified using a BCA Protein Assay Kit (Bio-Rad, Hercules, CA, USA). The proteins were digested using filter-aided sample preparation (FASP) and the peptide fraction was quantified. Peptides from each sample (100 μg) were labeled with TMTs (Thermo Fisher Scientific) according to the manufacturer’s instructions. The tryptic peptides were fractionated using a high-pH reverse-phase high-performance liquid chromatography (HPLC) system using a Thermo Betasil C18 column (10 cm, ID75 μm, 3 μm, C18-A2). Briefly, one unit of TMT reagent was thawed and reconstituted in acetonitrile. The peptide mixtures were then incubated for 2 h at room temperature and pooled, desalted, and dried by vacuum centrifugation. HPLC-MS/MS was performed using an Easy nLC system (Thermo Fisher Scientific) coupled to Q Exactive mass spectrometer (Thermo Scientific).

### Bioinformatic Analysis

The gene ontology (GO) annotation of differentially expressed proteins (DEPs) was performed by Blast2GO software (http://blast2go.com). Functional annotation was based on the online Kyoto Encyclopedia of Genes and Genomes (KEGG) Automatic Annotation Server database (http://www.kegg.jp/). A fold-change <0.83 or >1.2 with a *P*-value (Student’s *t*-test) <0.05 was selected as the cutoff criteria for the identification of DEPs. GO enrichment and KEGG pathway enrichment was performed based on the Fisher’s exact test with a *P*-value <0.05 and a false discovery rate (FDR) value <0.01.

### Western Blot Analysis

Total protein was extracted using RIPA lysis buffer containing protease inhibitors (APExBIO Technology, Houston, USA). Protein concentrations were determined using BCA Protein Assay Kit (Bio-Rad, Hercules, CA, USA). Then 10% SDS-PAGE gel electrophoresis was carried out using 50 μg of protein from each sample. After gel transfer, PVDF membranes were incubated with primary antibodies including rabbit anti-Bax (1:1,000, Proteintech, Wuhan, China), Bcl-2 (1:1,000, Proteintech, Wuhan, China), survivin (1:1,000, Proteintech, Wuhan, China), cleaved caspase-3 (1:1,000, Proteintech, Wuhan, China), cleaved caspase-9 (1:1,000, Proteintech, Wuhan, China), p21 (1:1,000, Proteintech, Wuhan, China), cyclin D1 (1:1,000, Proteintech, Wuhan, China), IRS1(1:1,000, Affinity, USA), p-PI3K (1:1,000, Affinity, USA), p-AKT (1:1,000, Affinity, USA), PI3K (1:1,000, Affinity, USA), and AKT (1:1,000, Affinity, USA) overnight at 4°C. After washing, membranes were further incubated with fluorochrome-labeled anti-rabbit IgG secondary antibody (1:10,000, Proteintech, Wuhan, China) for 1 h at room temperature. The membranes were imaged using the Odyssey Infrared Imaging System (LI-COR Biosciences, Lincoln, NE, USA), which was used to normalize relative expression level of each protein to GAPDH.

### Animal Experiments

The animal studies were conducted in accordance with the international standards-3R principle of animal welfare, and approved by the Experimental Animal Ethics Committee of the Fourth Hospital of Hebei Medical University. Approximately 5–6 weeks old Male BALB/c nude mice were obtained from the Beijing Vital River Laboratory Animal Technology (Beijing, China) and were housed at the Fourth Hospital of Hebei Medical University Experiment Animal Centre. Approximately 1 × 10^6^ HCT 116 cells suspended in 100 μl of PBS were subcutaneously injected into the right flank of each mouse. Tumor volume was measured every three days using a caliper and tumor volume was calculated by formula: tumor volume = (length × width^2^)/2. When the tumors’ volume reached 100 mm^3^, the mice were randomly divided into four groups (n = 6 per group). Control group (CON): mice were intraperitoneally injected with 0.9% physiological saline every two days. PPM group (PPM): mice were intraperitoneally injected with 3 mg/kg PPM every two days. Fluorouracil group (5-FU): mice were intraperitoneally injected with 10 mg/kg fluorouracil every two days. PPM + fluorouracil group (PPM + 5-FU): mice were intraperitoneally injected with a combination of PPM (3 mg/kg) and fluorouracil (10 mg/kg) every two days. Approximately 21 days after treatment, mice were sacrificed by spinal dislocation and the tumors were harvested and weighed. Blood were collected from eyeball and used for further analysis. The tumors were fixed in formalin and used for histological study.

### Histology and Immunohistochemistry

Six mice in each group were for pathologic examinations, included hematoxylin and eosin (HE) staining and immunohistochemistry (IHC) study. Tumor tissue were fixed, dehydrated and then embedded in paraffin. Slices were cut into 4 um and dewaxed and then dehydrated. HE staining was performed according to the protocol. Six xenografted tumors in each group were used for the quantification of the IHC studies. Immunohistochemistry (IHC) was carried out according to standard procedures. Primary antibodies of Bax (1:200, Proteintech, Wuhan, China), Bcl-2 (1:200, Proteintech, Wuhan, China), cleaved caspase-3 (1:200, Proteintech, Wuhan, China), IRS1 (1:200, Affinity, USA), p-PI3K (1:200, Affinity, USA), and p-AKT (1:200, Affinity, USA) were incubated overnight at 4°C. The staining intensity was scored as follows: 0 (negative), 1 (weak staining), 2 (moderate intense staining), or 3 (strong staining). The extent of the staining was scored based on the percentage of positive cells: 0 (no staining), 1 (1–10% staining), 2 (10–50% staining), and 3 (more than 50% staining). The final IHC score was obtained by multiplying the intensity and percentage scores as described previously (13).

### Blood Analysis

Routine blood test was performed by using a Mindray BC-6800Plus hematology analyzer (Mindray Biomedical Electronics, Shenzhen, China). Serum parameters of liver and kidney function were measured by Beckman Coulter AU5800 chemistry analyzer (Beckman Coulter, USA).

### Statistical Analysis

Data were analyzed using GraphPad Prism v5.0 and SPSS v21.0 and are presented as means ± standard error of the mean (SEM). One-way analysis of variance (ANOVA) was used for comparisons among multiple groups and repeated measure of ANOVA was used for analysis of tumor volume. For comparisons between two groups, the SNK-q test was used. *P <*0.05 was considered statistically significant.

## Results

### PPM Inhibits the Viability of CRC Cells

**Figure 1:**
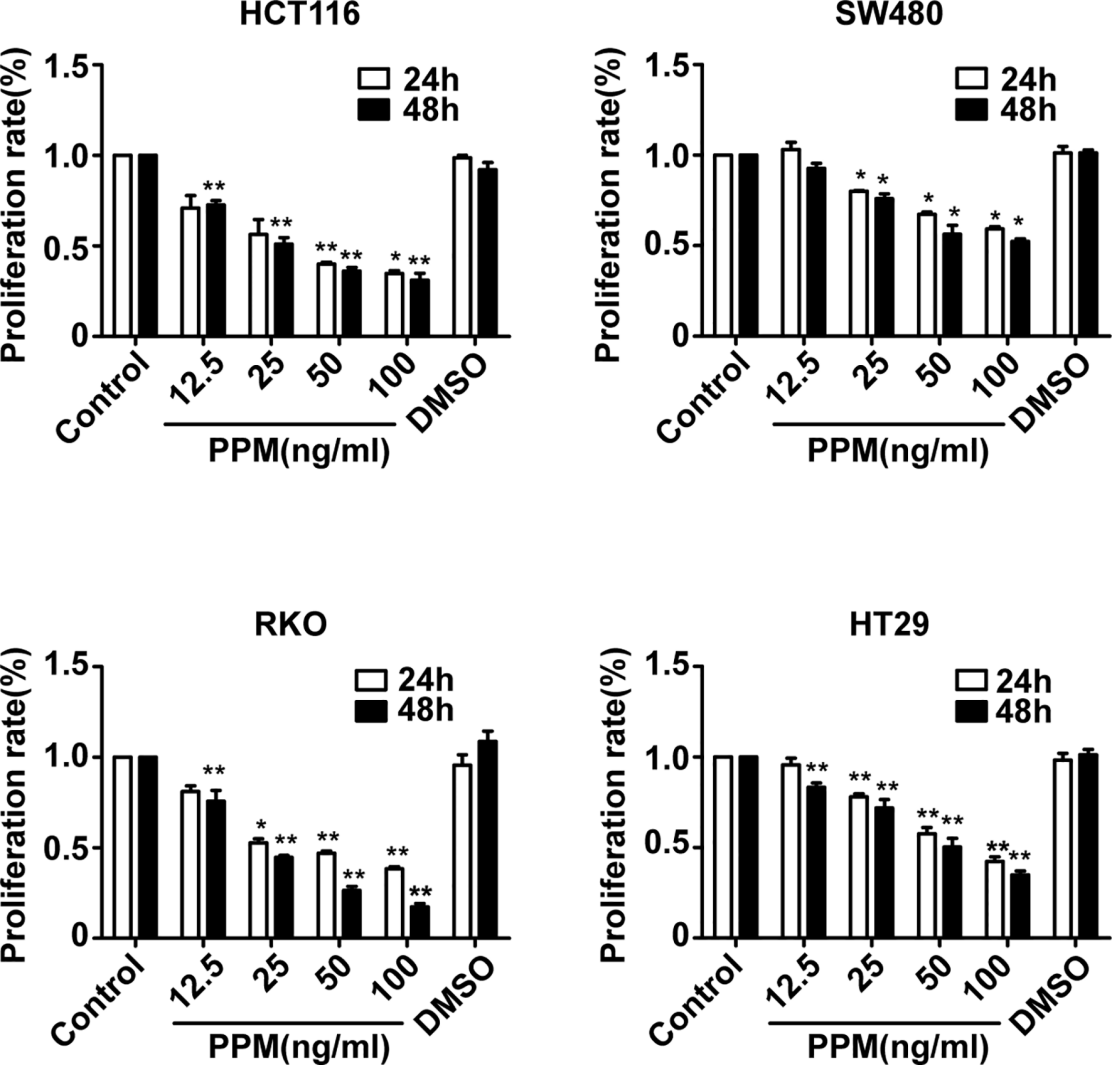
The effect of periplocymarin (PPM) on the viability of colorectal cancer cells. Colorectal cancer cells (HCT 116, SW480, RKO, and HT-29) were treated with different concentrations of PPM (0, 12.5, 25, 50, or 100 ng/ml) and 0.01% DMSO for 24 and 48 h. Cell viability was determined by CCK-8 assays. Data are shown as means ± SEM. *P < 0.05, **P < 0.01, *vs*. untreated control group.

To explore the effects of PPM on CRC cells, the viability of CRC cells was assessed by CCK-8 assay. The results showed that, the viability of HCT 116, RKO, HT-29, and SW480 cells were inhibited by PPM in a dose- and time-dependent manner (*P <*0.001) (**Figure 1**), indicating PPM may inhibit CRC cells proliferation. The IC_50_ values of PPM against HCT 116, RKO, HT-29, and SW480 cells at 24 h were 35.74 ± 8.20 ng/ml, 45.60 ± 6.30 ng/ml, 72.49 ± 5.69 ng/ml, and 112.94 ± 3.12 ng/ml, respectively. HCT 116 and RKO cell lines were more prone to the inhibition by PPM, therefore used for further analysis.

### PPM Induced Cell Apoptosis in CRC Cells

**Figure 2:**
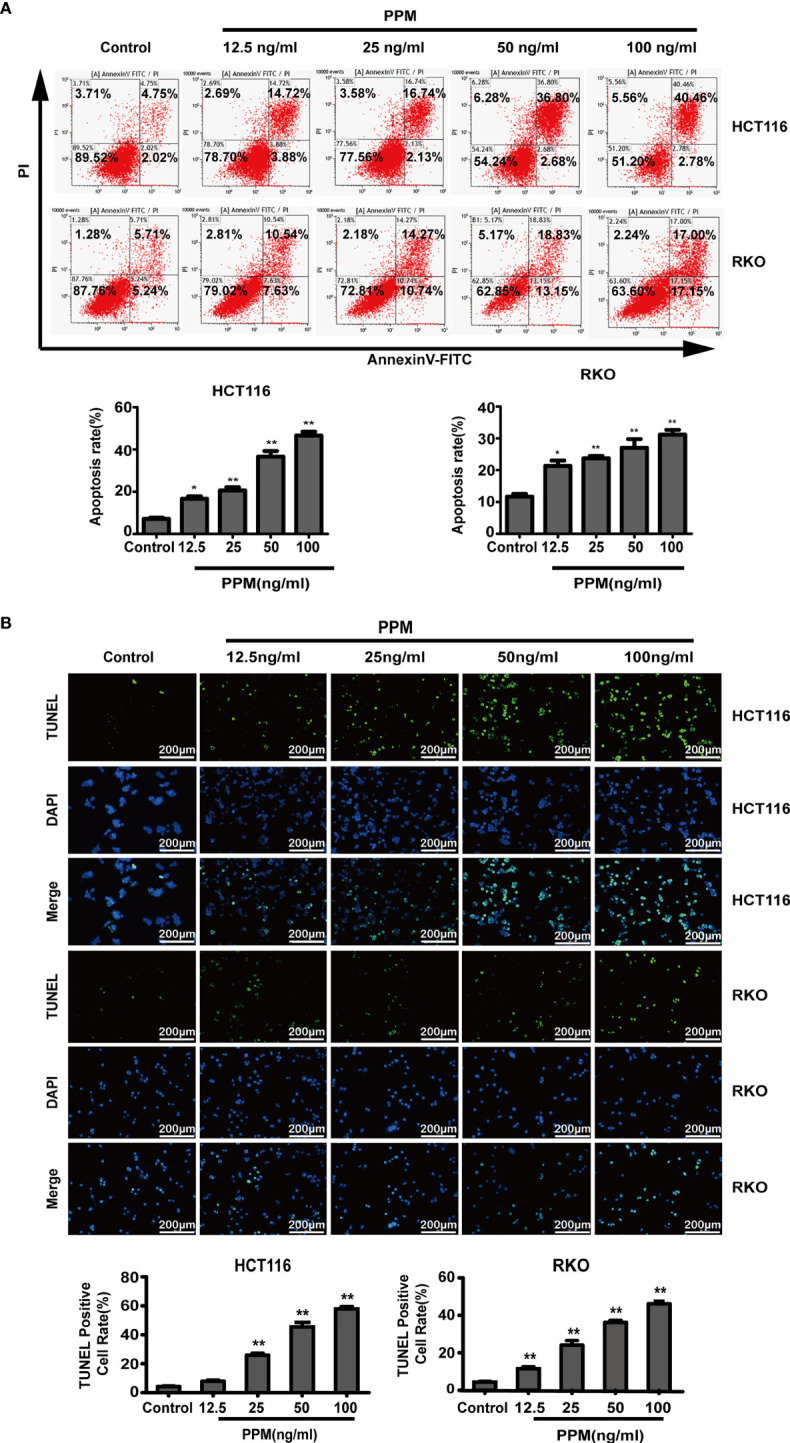
Periplocymarin (PPM) induces apoptosis in colorectal cancer cells. **(A)** Flow cytometric analysis of HCT 116 and RKO cells treated with different concentrations of PPM (0, 12.5, 25, 50, or 100 ng/ml) for 24 h. The cells were examined by Annexin V-FITC staining. **(B)** TUNEL staining of HCT 116 and RKO cells treated with PPM (0, 12.5, 25, 50, or 100 ng/ml) for 24 h. Apoptotic cells (TUNEL-positive cells) exhibit green fluorescence. Scale bar, 200 μm. Data are shown as means ± SEM. *P < 0.05, **P < 0.01, *vs*. untreated control group.

To clarify the effect of PPM on the apoptosis of CRC cells, HCT 116 and RKO cells were treated with different concentration of PPM for 24 h, and the number of apoptotic cells was detected by flow cytometry. Data indicated that, compared to control, PPM treatment caused significantly increased apoptotic ratio in both HCT 116 and RKO cells in a dose dependent manner (**Figure 2A**). Similar results were also exhibited in TUNEL staining, which indicated that PPM treatment caused increased TUNEL-positive CRC cell ratio (**Figure 2B**).

**Figure 3:**
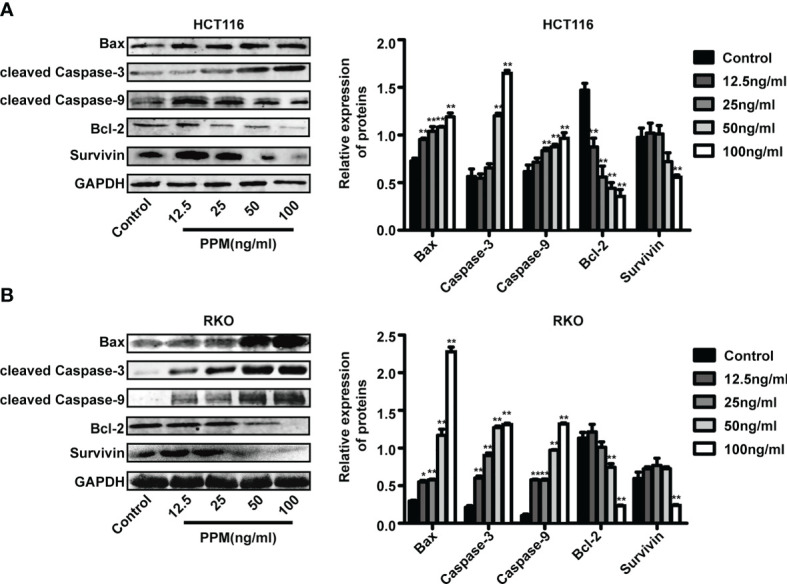
Periplocymarin (PPM) altered gene expression involved in apoptosis. Western blot analysis of Bax, Bcl-2, cleaved caspase-3, cleaved caspase-9, and survivin protein levels in HCT 116 **(A)** and RKO cells **(B)** treated with different concentrations of PPM (0, 12.5, 25, 50, or 100 ng/ml) for 24 h. Values are shown as means ± SEM. *P < 0.05, **P < 0.01, *vs*. untreated control group.

Previous studies have found that the increase in the Bax/Bcl-2 ratio can lead to cytochrome c release and the subsequent cleavage and activation of caspase-3 and caspase-9, which can result in the degradation of intracellular substrates and apoptosis (14). This study detected these apoptosis related proteins. Data from western blot has shown that, PPM treatment caused pro-apoptotic gene expression profile, which exhibited increased pro-apoptotic gene expression such as Bax, cleaved caspase-3 and cleaved caspase-9, while decreased anti-apoptotic gene expression such as survivin and Bcl-2 (**Figures 3A, B** **)**. Therefore, PPM may cause CRC cell apoptosis by targeting relative gene expression.

### PPM Induced Cell Cycle Arrest

**Figure 4:**
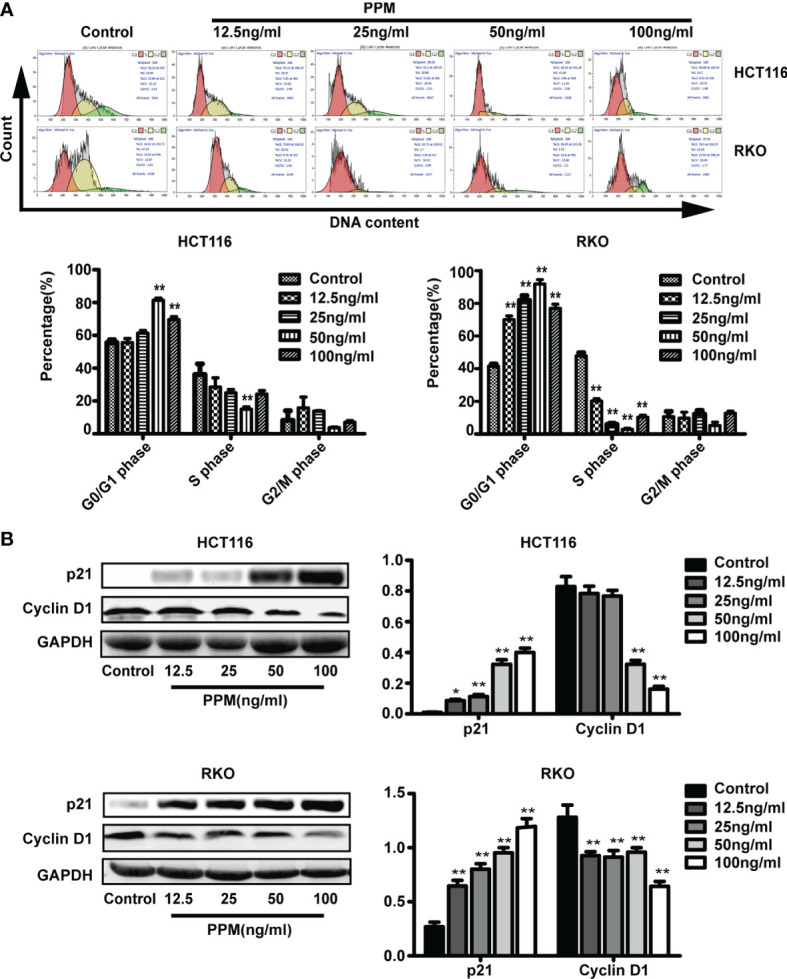
Periplocymarin (PPM) induces cell cycle arrest in colorectal cancer cells. **(A)** PPM induced G0/G1 cell cycle arrest in HCT 116 and RKO cells as determined by flow cytometry. **(B)** The protein expression of cyclin D1 was decreased and that of P21was increased with PPM treatment. Values are shown as means ± SEM. *P < 0.05, **P < 0.01, *vs*. untreated control group.

We further examined whether PPM have regulated the cell cycle. Data from flow cytometry has indicated that, compared to control, PPM treatment may cause significantly increased ratio of cells in G0/G1 phase while decreased ratio of cells in S phase, indicating PPM may promote cycle arrest in CRC cells (**Figure 4A**) and the maximum effect of PPM observed in the present study was at concentration of 50 ng/ml.

Cyclin D1 and p21 are both involved in cell cycle regulation. Data from the present study has found that, compared to control, PPM treatment may increase p21 expression in a dose-dependent manner in both cell lines (**Figure 4B**). PPM treatment led to decreased cyclin D1 gene expression at concentration of 50 ng/ml onward in HCT 116 cells, while this inhibitory effect can be found in all PPM-treated RKO cells. The maximal effect of PPM on cyclin D1 expression was noted at 100 ng/ml in both cell lines.

### DEPs Identification

**Figure 5:**
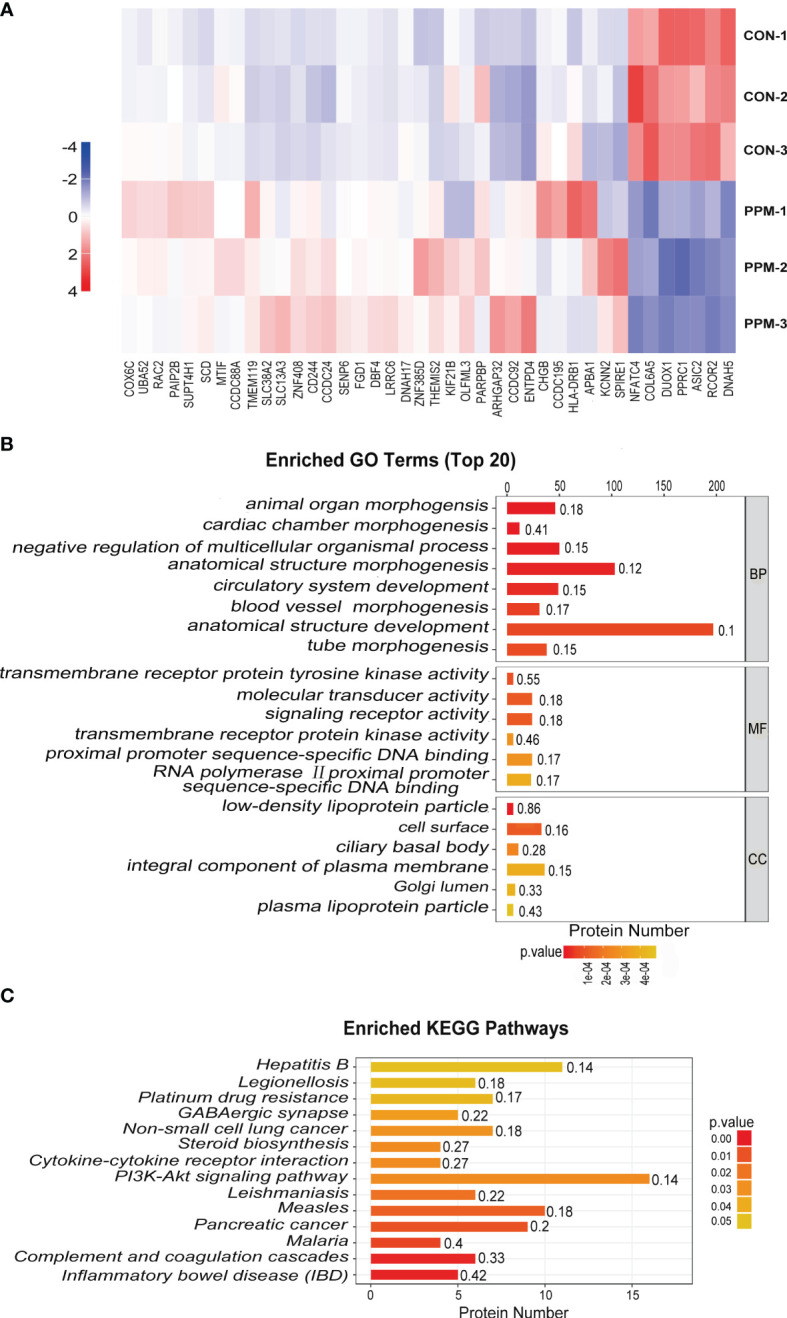
Quantitative proteomic analysis of control and PPM-treated HCT 116 cells and bioinformatic analyses of the differentially expressed proteins (DEPs). **(A)** Hierarchical clustering of DEPs between the control and PPM-treated groups. The heatmap is a visualized demonstration of the protein distribution in different samples. Columns represent the proteins and rows represent the groups of cells. The red color represents high expression and blue color represents low expression. A total of 33 of the upregulated proteins and seven of the downregulated proteins showed >2 fold-changes with *P <* 0.05. **(B)** Top 20 enriched GO terms using Fisher’s exact test for the biological process (BP), molecular function (MF), and cellular component (CC) categories. The vertical axis represents GO terms in each category; the horizontal axis demonstrates the protein number of each item. The numbers beside the bars are enrichment factors, which represent the significance and reliability of proteins enriched in each item. **(C)** Enriched KEGG pathways associated with the DEPs. The color of the bar represents the *P*-value calculated using Fisher’s exact test.

Tag-based (TMT) quantitative proteomics technology was performed to detect proteins of CRC cells under PPM treatment. Of those DEPs, 286 were found upregulated and 293 were downregulated. Among them, 33 of the upregulated proteins and seven of the downregulated proteins showed >2 fold-changes with *P <*0.05 (**Figure 5A** and **Table 1**). The most enriched GO terms were annotated as transmembrane receptor protein tyrosine kinase activity in the molecular function category (GO: 0004714, six proteins, *P* = 8.99E−05), low-density lipoprotein particle in the cellular compartment category (GO: 0034362, six proteins, *P* = 1.81E−06), and animal organ morphogenesis in regard to the biological process category (GO: 0009887, 46 proteins, *P* = 1.76E−07) (**Figure 5B**). KEGG pathway enrichment analysis was performed to check pathways associated with the DEPs. The results showed that 14 KEGG pathways were significantly enriched based on the number of proteins with *P <*0.05. The top seven enriched pathways were Inflammatory bowel disease (five proteins, *P* = 0.001687), Complement and coagulation cascades (six proteins, *P* = 0.002066), Malaria (four proteins, *P* = 0.006051), Pancreatic cancer (proteins, *P* = 0.010368), Measles (10 proteins, *P* = 0.013437), Leishmaniasis (six proteins, *P* = 0.018835), and the PI3K/AKT signaling pathway (16 proteins, *P* = 0.028105). DEPs were more associated with the PI3K/AKT signaling pathway which might participate in the proapoptotic effects of PPM on CRC cells (**Figure 5C**).

**Table 1:** DEPs between PPM-treated groups and control groups in HCT 116 cells.

Accession	Protein Name	Gene Name	Fold chang	p value
Up-regulated proteins				
Q8N4L8	Coiled-coil domain-containing protein 24	CCDC24	6.81	0.04
Q9GZR1	Sentrin-specific protease 6	SENP6	4.71	0.02
Q3V6T2	Girdin	CCDC88A	4.63	0.03
P98174	FYVE, RhoGEF and PH domain-containing protein 1	FGD1	4.44	0.04
Q9H6B1	Zinc finger protein 385D	ZNF385D	3.86	0.01
Q9Y227	Ectonucleoside triphosphate diphosphohydrolase 4	ENTPD4	3.35	0.02
Q8N511	Transmembrane protein 199	TMEM199	3.30	0.04
P05060	Secretogranin-1	CHGB	3.29	0.01
Q9H2S1	Small conductance calcium-activated potassium channel protein 2	KCNN2	3.20	0.00
Q5TEJ8	Protein THEMIS2	THEMIS2	3.19	0.02
Q30167	HLA class II histocompatibility antigen, DRB1-10 beta chain	HLA-DRB1	3.07	0.04
A0A1B0GUA6	Putative coiled-coil domain-containing protein 195	CCDC195	3.08	0.01
Q96QD8	Sodium-coupled neutral amino acid transporter 2	SLC38A2	3.00	0.00
Q02410	Amyloid-beta A4 precursor protein-binding family A member 1	APBA1	2.97	0.03
Q9NRN5	Olfactomedin-like protein 3	OLFML3	2.73	0.02
A8TX70	Collagen alpha-5(VI) chain	COL6A5	2.69	0.01
Q9NRD9	Dual oxidase 1	DUOX1	2.52	0.00
Q9NWS1	PCNA-interacting partner	PARPBP	2.51	0.02
Q86X45	Protein tilB homolog	LRRC6	2.50	0.04
A7KAX9	Rho GTPase-activating protein 32	ARHGAP32	2.33	0.01
Q53HC0	Coiled-coil domain-containing protein 92	CCDC92	2.29	0.02
Q9UBU7	Protein DBF4 homolog A	DBF4	2.26	0.03
Q8IZ40	REST corepressor 2	RCOR2	2.25	0.00
Q8WWT9	Solute carrier family 13 member 3	SLC13A3	2.24	0.03
Q08AE8	Protein spire homolog 1	SPIRE1	2.19	0.01
Q9H9D4	Zinc finger protein 408	ZNF408	2.15	0.00
Q9UFH2	Dynein heavy chain 17, axonemal	DNAH17	2.13	0.04
Q16515	Acid-sensing ion channel 2	ASIC2	2.12	0.00
Q8TE73	Dynein heavy chain 5, axonemal	DNAH5	2.12	0.00
O75037	Kinesin-like protein KIF21B	KIF21B	2.10	0.02
Q14934	Nuclear factor of activated T-cells, cytoplasmic 4	NFATC4	2.06	0.01
Q5VV67	Peroxisome proliferator-activated receptor gamma coactivator-related protein 1	PPRC1	2.02	0.00
Q9BZW8	Natural killer cell receptor 2B4	CD244	2.02	0.01
Down-regulated proteins				
O00767	Acyl-CoA desaturase	SCD	0.49	0.00
P04733	Metallothionein-1F	MT1F	0.47	0.01
P09669	Cytochrome c oxidase subunit 6C	COX6C	0.45	0.04
P62987	Ubiquitin-60S ribosomal protein L40	UBA52	0.45	0.01
P15153	Ras-related C3 botulinum toxin substrate 2	RAC2	0.45	0.04
Q9ULR5	Polyadenylate-binding protein-interacting protein 2B	PAIP2B	0.41	0.04
P63272	Transcription elongation factor SPT4	SUPT4H1	0.41	0.04

### PPM Impairs PI3K/AKT Signaling Pathway

**Figure 6:**
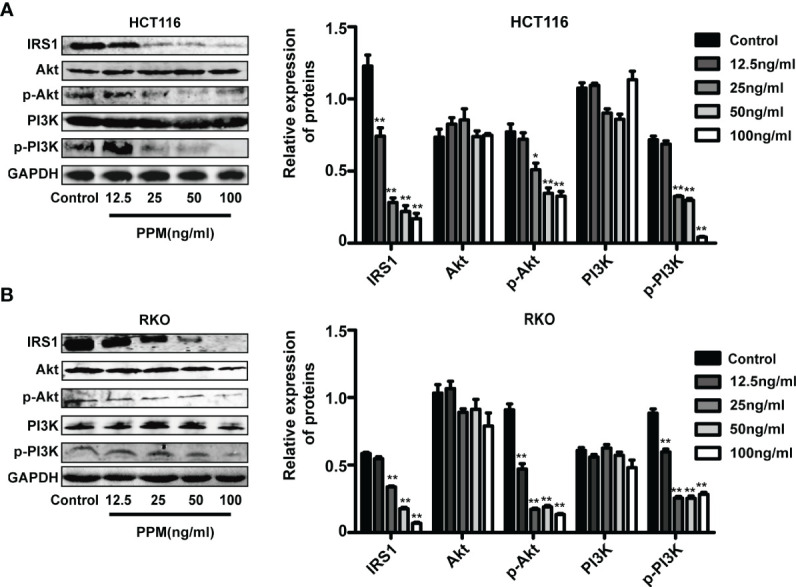
Periplocymarin (PPM) modulates gene expression involved in PI3K/AKT signaling pathway. PPM treatment altered the expression levels of IRS1, PI3K, AKT, p-PI3K, and p-AKT in HCT 116 **(A)** and RKO cells **(B)**. Values are shown as means ± SEM. *P < 0.05, **P < 0.01, *vs*. untreated control group.

PI3K/AKT signaling pathway activation has been found promotes tumor progression and cell survival by inhibition of apoptosis (15). PI3K and AKT are both the downstream target genes of IRS1 (16) and the phosphorylation forms are the active ones. In the present study, consistent with TMT-based results, PPM treatment caused significantly decreased IRS1 expression in HCT and RKO cells compared to control cells in almost a PPM concentration-dependent manner. Although gene expression of PI3K and AKT were not altered, both p-PI3K and p-AKT were significantly decreased by PPM treatment. Therefore, PPM treatment may impair PI3K/AKT signaling pathway, contributing to increased CRC cell apoptosis ratio (**Figures 6A, B** **)**. The mass spectrometry proteomics data have been deposited to the ProteomeXchange Consortium (http://proteomecentral.proteomexchange.org) *via* the iProX partner repository (17) with the dataset identifier PXD029172.

### The Effects of PPM on Blood Parameters

To examine the safety of PPM, blood parameters were checked in the nude mice. Data indicated that, compared to CON animals, PPM treatment caused no alterations in blood cells, hepatic and renal functional parameters. However, 5-FU treated animals exhibited lower WBC number and higher ALT and AST levels compared to CON animals (*P* all <0.05), indicating hypoleukemia and liver functional impairment. Only increased UN level was found in PPM + 5-FU group, indicating that 5-FU combined with PPM might cause renal functional impairment. Meanwhile, the WBC count and ALT, AST levels in PPM + 5-FU group showed no difference compared to those in CON group, suggesting PPM might alleviate the adverse effects of 5-FU on hepatic function and WBC (**Table 2**).

**Table 2:** The effects of periplocymarin (PPM) and 5-FU on blood cell counts and liver and kidney function.

	WBC (×10^9^/L)	RBC (×10^12^/L)	PLT (×10^9^/L)	ALT (U/L)	AST (U/L)	CREA (μmol/L)	UN (mmol/L)
**CON**	0.64 ± 0.06	3.33 ± 0.04	309.67 ± 11.57	10.63 ±1.20	57.40 ± 10.06	2.27 ± 0.46	2.47 ± 0.09
**5-FU**	0.32 ± 0.04*	3.37 ± 0.11	276.00 ± 26.01	47.53 ±13.43*	113.67 ± 20.07*	4.67 ± 0.44	6.77 ± 1.26
**PPM**	0.68 ± 0.06	3.42 ± 0.01	327.33 ± 16.76	8.00 ± 1.66	37.03 ± 0.58	2.33 ± 0.18	2.20 ± 0.17
**PPM + 5-FU**	0.54 ± 0.08	3.51 ± 0.09	336.00 ± 7.21	25.13 ± 4.0	77.03 ± 4.71	3.93 ± 1.66	5.23 ± 0.27*

WBC, white blood cell; RBC, red blood cell; PLT, platelet; ALT, alanine aminotransferase; AST, aspartate aminotransferase; CREA, creatinine; UN, urea nitrogen.

Data were present as mean±SEM. N=3 per/group.

*P < 0.05 vs. CON group.

### The Effects of PPM on Colorectal Tumor Formation *In Vivo*

**Figure 7:**
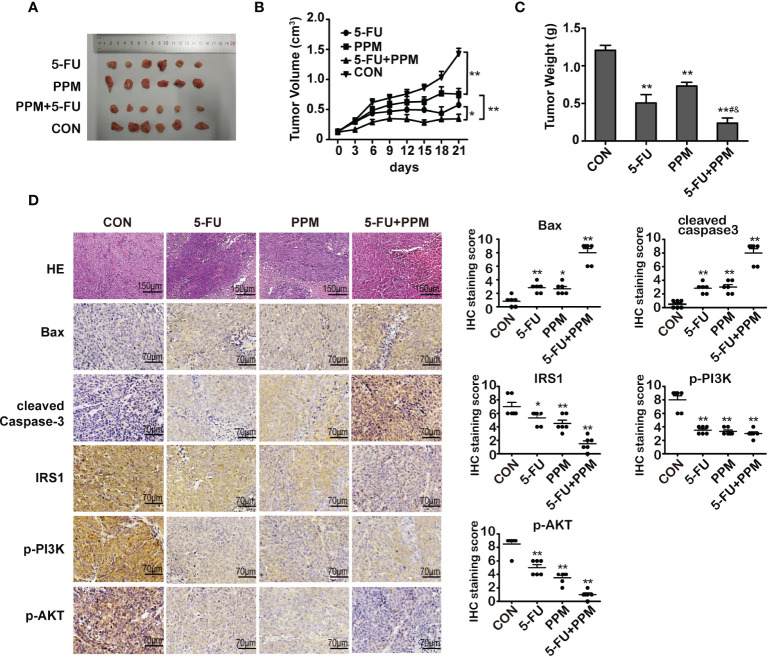
The effect of periplocymarin (PPM) on colorectal cancer cells in nude mice. **(A)** Images of tumors derived from mice in each group. **(B)** Tumor volumes were calculated in each group every 3 days from days 1 to 21. **(C)** Tumor weights were measured on day 21. **(D)** Hematoxylin/eosin-stained sections of tumor tissues derived from mice in each treatment group were presented. Magnification: 200×. Scale bar, 150 μm. The expression of Bax, cleaved caspase-3, IRS1, p-PI3K, and p-AKT in tumor tissues of mice from each treatment group was examined by immunohistochemistry. Magnification: 400×. Scale bar, 70 μm. *P < 0.05, **P < 0.01, *vs*. untreated control group. ^#^ *P <* 0.05, *vs*. 5-FU treatment group. ^&^ *P <* 0.05, *vs*. PPM treatment group. n =6 per group.

To explore the effects of PPM on CRC cells *in vivo*, BALB/c nude mice with HCT 116 cells xenografted tumor were treated. Data from the present experiment has shown that, compared to control animals, PPM, 5-FU and PPM + 5-FU treatment all could impair tumor growth. There were no statistical differences in tumor volume between 5-FU and PPM-treated group (**Figures 7A, B** **)**. At day 21, compared to control group, the weights of tumors were found reduced in PPM, 5-FU and PPM + 5-FU treated animals. There were no statistical differences in tumor weight between 5-FU and PPM-treated group (**Figure 7C**). The tumor weight in PPM + 5-FU-treated group was even lower when compared to PPM and 5-FU-treated groups separately (**Figure 7C**), indicating the anti-tumor effect might be exaggerated in PPM and 5-FU combination treatment.

Gene expression involved in cell apoptosis were also measured by immunohistochemistry, which indicated that PPM, 5-FU, and PPM + 5-FU treatment all led to increased gene expression of Bax and cleaved caspase-3 while decreased expression of IRS1, p-PI3K, and p-AKT (**Figure 7D**), indicating PPM may perform pro-apoptotic effects *in vivo*. Except p-PI3K, the most manifest effects in those gene expressions were found in PPM + 5-FU-treated animals, suggesting the pro-apoptotic effects might also be exaggerated by PPM and 5-FU combination therapy strategy.

## Discussion

Colorectal cancer (CRC) is one of the most common malignancies worldwide, and is the second leading cause of tumor death (18). For some stage IV colon and rectal cancers with metastases, radiotherapy and chemotherapy therapy are the main therapy strategies so far. However, the chemotherapy drugs are effective but combined with unwanted toxicity and side effects (5).

It has been found that, cardiac glycosides (CG) may have the ability to induce tumor cell death *in vitro* (19–21). Retrospective clinical analyses revealed that, CG digoxin administration during chemotherapy may improve overall survival in cohorts of breast, colorectal, head and neck, and hepatocellular carcinoma patients (22). Moreover, elevated plasma digitoxin levels have been found previously correlate with reduced incidence of leukemia, lymphoma, and kidney/urinary tract tumors in patients (23). PPM, a cardiac glycoside isolated from *Cortex Periplocae*, has been used for chronic congestive heart failure treatment due to its effect of strengthen myocardial contractility (24). Previous studies performed in some tumor cells, such as PC3, U937, MCF-7, and SMMC-7721, have found that, PPM has potent anti-cancer effect by inhibiting cells growth and promoting apoptosis (10, 12), suggesting PPM applications in anti-cancer treatment.

Most anti-cancer therapies trigger apoptosis to eliminate malignant cells (25, 26). Previous study indicated that PPM inhibited prostate cancer cell line PC3 growth by the activation of caspase-dependent apoptotic pathways (10). PPM also could sensitize U937 cells to TNF-related apoptosis, which was more quickly than the ouabain (10). In the present experiment, we have found that PPM promoted CRC cell apoptosis *in vitro*. Our study showed PPM treatment led to a significant reduction in the viability of several CRC cell lines and increased the rate of apoptosis in HCT 116 and RKO cells in a dose-dependent manner. In addition, we are first to discover that PPM could trigger an increase of expression profile for the pro-apoptotic proteins Bax, cleaved caspase-3, and cleaved caspase-9, and a decrease in those of the anti-apoptotic proteins Bcl-2 and survivin, contributing to increased apoptotic ratio in CRC cell (14, 27). However, mechanisms underlying PPM modulating apoptosis is still quite limited and detailed studies needs to be further performed.

Cell cycle disruption has been found in pathogenesis of tumors (28). In this study, we observed that PPM treatment could cause significantly increased ratio of cells in G0/G1 phase while decreased ratio of cells in S phase, indicating PPM promoted cycle arrest in CRC cells. But the maximum effect of PPM observed was at concentration of 50 ng/ml, suggesting that its effect on cell cycle does not enhance as the drug concentration increases when greater than 50 ng/ml. Protein p21 is a negative regulator of G1/S transition (29), and downregulation of p21 is involved in tumor promotion in various cancers (30, 31). Oncogene cyclin D1 has been found overexpressed in human cancers (32) and implicated in many activities, such as cell cycle promotion, chromosomal instability, mitochondrial function and cellular senescence (33). Previous studies have found that cyclin D1 binds and sequesters p21, thereby allowing the progression from G1 to S phase (34). In the present study, we found that PPM treatment increased p21 and decreased cyclin D1 gene expression, indicating that PPM reduced CRC cell proliferation mainly by causing cell cycle arrests, and this effect might be achieved by increasing p21 and reducing cyclin D1 expression. Similar results have also been found in some other traditional Chinese medicine, such as curcumol and pigallocatechin-3-gallate (35, 36). But the maximal effect of PPM on p21 and cyclin D1 expression was noted at 100 ng/ml, indicating that there might be other proteins involved in the cell cycle regulation of PPM treatment, which needs further research.

Quantitative proteomic techniques have been widely applied due to their ability to reveal the dynamics of protein expression and protein-protein interactions from a global perspective, which greatly help to understand the gene function in cellular processes (37). In this study, the proteomic approach has been introduced to identify PPM treatment-associated DEPs. A total of 6,645 proteins were identified, of these, 539 were found to be differentially expressed. Gene Ontology (GO) analysis suggests these proteins have important functions in various metabolic processes, (e.g., transmembrane receptor protein tyrosine kinase activity, signaling receptor activity, and molecular transducer activity). PI3K/AKT signaling pathway was selected for further mechanism research owing to its high enrichment factor and more DEPs associated according to the KEGG analysis. At the same time, the results also showed that IRS1, as the upstream of PI3K, was down-regulated after PPM treatment.

PI3K/AKT signaling pathway plays an important role in tumor cell growth, proliferation and survival by regulating apoptosis-related genes (15). Elevated level of phosphorylated PI3K and AKT were found in human CRC samples, which was correlated with a poor disease outcome (38). Activated PI3K/AKT signaling pathway has been suggested favorable for cells survival through inhibiting apoptosis and impair cell cycle arrest by targeting downstream genes, including Bcl-2 family, caspase family, survivin, p21, and cyclin D (39–43). Inhibition of PI3K/AKT signaling pathway has been found effective in inhibition of tumor cells of different tissues (44–46). IRS1, upstream of PI3K and major substrate of insulin, insulin-like growth factors and cytokine signaling, plays an important role in mediating apoptosis, cell differentiation, and cell transformation (47). IRS1 is constitutively activated in a variety of solid tumors, namely, CRC, breast cancers, leiomyomas, Wilms tumors, rhabdomyosarcomas, liposarcomas, leiomyosarcomas, and adrenal cortical carcinomas (48). In the present study, we found that PPM reduced IRS1, p-PI3K, p-AKT gene expression and caused impaired PI3K/AKT signaling pathway in CRC cells, which might lead to increased apoptotic ratio and promoted cell cycle arrest.

Also, in the present experiment, we have checked the effect and safety of PPM in animal models. We have found that, PPM performed comparable anti-tumor effect with 5-FU, but with less negative effects in white cells and liver function. Studies about PPM on blood, renal and hepatic function is little. It has been found that the toxicity of *Cortex Periplocae* is mainly cardiac glycoside poisoning (49). However, previous study has found that, *Cortex Periplocae* extract may reverse the white blood cell decline caused by cyclophosphamide (50). Wan et al. (51) found that periplocoside A, another nature product compound isolated from *Cortex Periplocae*, may prevent liver damage caused by concanavaline A. Intriguingly, the PPM + 5-FU combination therapy strategy seemed more effective in tumor suppression than single drug treatment, and only slightly renal function impairment was observed, indicating PPM might be added to other traditional anti-tumor therapy, more favorable effects would be achieved with less side effects. However, long-term *in vivo* effect of PPM still needs to be investigated. In addition, the cardiovascular safety of PPM is still needed to be warranted.

**Figure 8:**
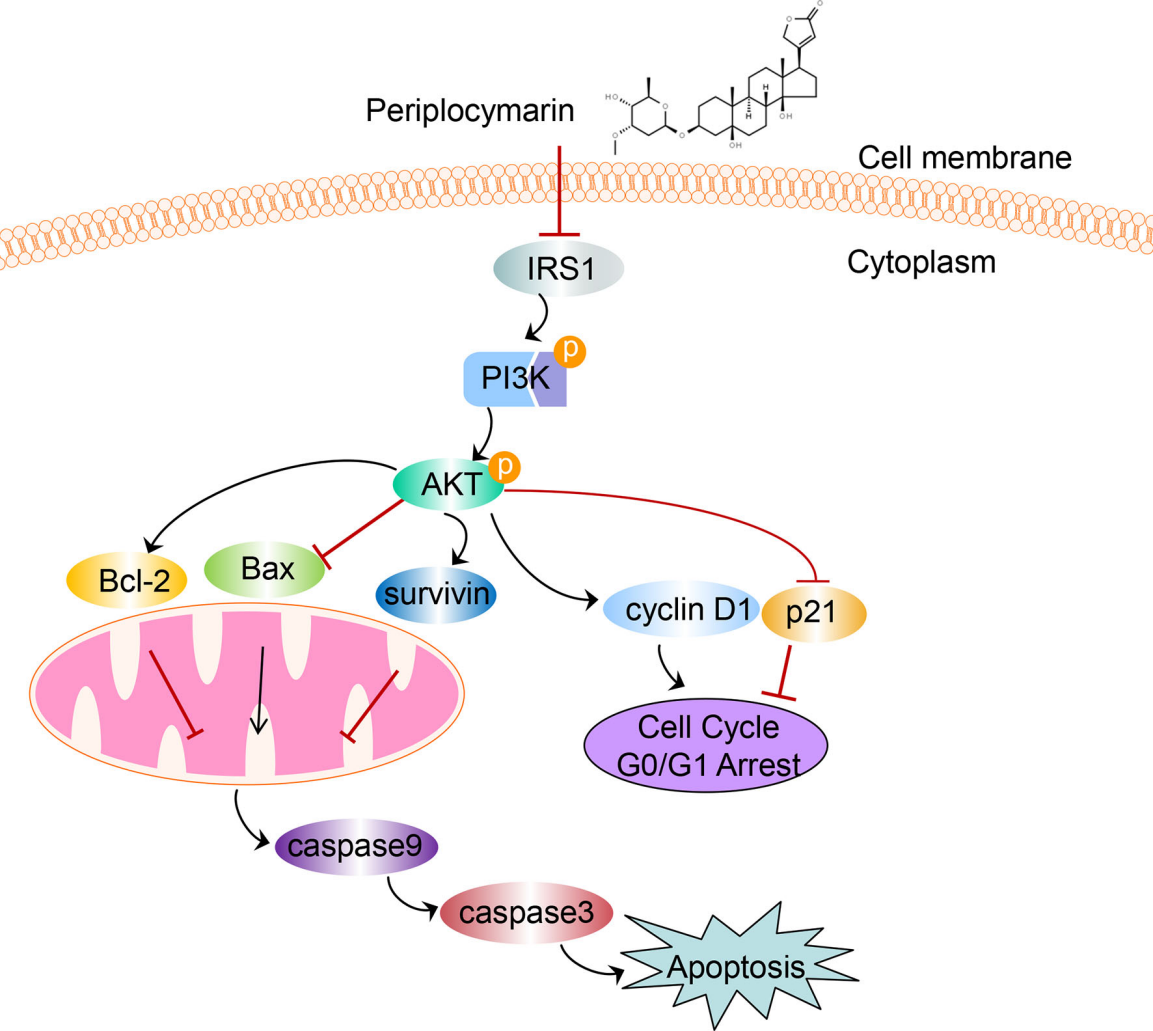
The schematic diagram depicts the mechanism of PPM-induced apoptosis and cell cycle arrest *via* impairing PI3K/AKT signaling pathway in CRC cells. We supposed that PPM may inhibit the expression of IRS1, p-PI3K, and p-AKT and modulate the expression of Bcl-2 family, survivin, p21, and cyclin D1, which further promotes apoptosis and cell cycle arrest in G0/G1 phase of CRC cells.

In conclusion, data from this experiment indicated that PPM performed anti-tumor effects both *in vivo* and *in vitro*. Inhibited PI3K/AKT signaling pathway might be involved in this process. A diagram for the mechanism of PPM-induced apoptosis and cell cycle arrest *via* impairing PI3K/AKT signaling pathway in CRC cells is shown in **Figure 8**. PPM is a promising chemotherapeutic drug for CRC treatment but extended safety evaluation should be performed.

## Data Availability Statement

The original contributions presented in the study are included in the article/supplementary material. Further inquiries can be directed to the corresponding authors.

## Ethics Statement

The animal study was reviewed and approved by the Experimental animal ethics committee of the Fourth Hospital of Hebei Medical University.

## Author Contributions

GW and LZ conceived the study. YC, SD, and CZ performed the experiment. JH and XH analyzed experimental results. HM, BL, and ZM provided advice on the study design and data interpretation. YC and FW wrote the manuscript. All authors contributed to the article and approved the submitted version.

## Funding

This work was supported by the Natural Science Foundation of China (Grant No. 81772550), Natural Science Foundation precision medicine joint project of Hebei Province, China (Grant No. H2020206485), Department of science and technology key project of Hebei Province, China (Grant No. 206Z7705G) and Precision Medicine Joint Fund Cultivation Project of Hebei Province, China (Grant No. H2021206253).

## Conflict of Interest

The authors declare that the research was conducted in the absence of any commercial or financial relationships that could be construed as a potential conflict of interest.

## Publisher’s Note

All claims expressed in this article are solely those of the authors and do not necessarily represent those of their affiliated organizations, or those of the publisher, the editors and the reviewers. Any product that may be evaluated in this article, or claim that may be made by its manufacturer, is not guaranteed or endorsed by the publisher.
